# Increased incidence of second primary malignancy in patients with malignant astrocytoma: a population-based study

**DOI:** 10.1042/BSR20181968

**Published:** 2019-06-14

**Authors:** Wenming Wang

**Affiliations:** Department of Traditional Chinese Medicine, Beijing Tian Tan Hospital, Capital Medical University, 119 South 4th Ring West Road, Beijing 100070, China

**Keywords:** astrocytoma, multiple primaries-standardized incidence ratio, nomogram, second primary malignancy, SEER

## Abstract

We identified patients diagnosed with malignant astrocytoma (MA) as the first of two or more primary malignancies between 1973 and 2015 from Surveillance, Epidemiology and End Results (SEER) database. Multiple primaries-standardized incidence ratio (MP-SIR) was calculated to quantitate the risk of second primary malignancy (SPM). We further identified the risk factors of developing SPM and factors affecting overall survival (OS) in MA patients with SPM. Our results revealed that overall risk of SPM among MA patients was significantly higher than that in general population (SIR: 1.09, 95% confidence interval (CI): 1–1.18, *P*<0.05). Specific sites where the risk of SPM increased included salivary gland, bone and joints, soft tissue including heart, brain, cranial nerves other nervous system, thyroid, acute non-lymphocytic leukemia and acute myeloid leukemia. Overall risk of SPM in patients aged ≤29 and 30–59 years significantly increased (4.34- and 1.41-fold respectively). Whereas patients aged ≥60 years had a significantly decreased risk of SPM. Patients in the group of latency at 36–59, 60–119 and ≥120 months carried significantly increased overall risk of SPM. Multivariate analysis revealed that age, race, marital status, WHO grade, differentiated grade of cancer tissues, latency was independent predictor of OS in MA patients with SPM, which were all selected into the nomogram. The calibration curve for probability of survival showed good agreement between prediction by nomogram and actual observation. In conclusion, MA survivors should be advised of their increased risk for developing certain cancers in their lifetime. Our study had clinical implications for the surveillance of MA survivors at risk of developing SPM.

## Introduction

Cancer survivors have been increasing due to the improvement of diagnostic modalities and treatment of cancer. Approximately 18 million cancer survivors are expected by 2022 [1]. It is important to figure out the long-term effects of cancer and its treatment among cancer survivors. Second primary malignancy (SPM) is one of the most serious long-term complications in the population of cancer survivors. Previous studies have verified significantly increased risk of SPM after treatment of several primary cancers [2–5].

The incidence of primary brain tumors has been increasing over the past 30 years, especially in elderly people [6]. Astrocytoma, derived from astrocytic glial cells, accounts for more than 60% of all primary brain tumors [7,8]. According to the current WHO grading system of astrocytoma, which is based on the histopathological characteristics of the underlying tumor tissue, diffuse infiltrating astrocytoma is graded as low-grade diffuse astrocytoma (DA, grade II), anaplastic astrocytoma (AA, grade III) and glioblastoma (GBM, grade IV) in the order of increasing malignancy [9]. The median overall survival (OS) for patients with DA, AA and GBA is 6–8 years, 2–3 years and 10–20 months, respectively [10–15]. Multidisciplinary treatment modalities combined surgery, chemotherapy, radiation and targeted therapy have yielded increased survival in patients with astrocytoma [16].

The number of cancer survivor patients with astrocytoma is increasing. Additionally, study on the risk of developing SPM in astrocytoma survivors is still lacking. The current study aimed to evaluate the overall and site-specific risk of developing SPM in patients with malignant astrocytoma (MA). And to investigate the factors affecting OS in MA patients with SPM by using the National Cancer Institute’s Surveillance, Epidemiology and End Results (SEER) database.

## Methods

### Population

The study population was derived from the SEER database. Dataset of incidence-SEER 9 Regs Research Data, November 2017 Sub (1973–2015) <Katrina/Rita population adjustment> was used for the analysis of multiple primaries-standardized incidence ratio (MP-SIR). Case listing was based on dataset of incidence-SEER 18 Regs Research Data + Hurricane Katrina Impacted Louisiana Cases, November 2017 Sub (1973–2015 varying). All patients in the present study were diagnosed with MA by positive histology between 1973 and 2015. Only cases where MA was the first of the two or more primary malignancies were selected. Patients were excluded if the diagnosis of MA was made at autopsy or in the death certificate.

### Definition of SPM

SEER definition of SPM took the following factors into account: the site, the behavior, histology and date of diagnosis of the second malignancy. As well as the laterality if it was a paired organ [17]. According to the SEER definition of SPM, the criteria for patients with SPM after the diagnosis of MA were listed as following: (i) a new tumor that contained the same histology as the previous cancer and was diagnosed within 2 months of the previous cancer was considered as a single primary. (ii) Simultaneous multiple tumors with the same histologic type within the brain were regarded as a single tumor, and even when different tumors had different behavior codes. (iii) Multiple tumors with the same histologic type appearing in the brain and in a different site were regarded as multiple primary malignancies unless stated as metastatic tumors. (iv) Multiple tumors of different histologic types within the brain and in a different site were counted as multiple primary malignancies regardless of time.

### Statistical analysis

The risk of SPM was presented as standardized incidence ratio (SIR) and its 95% confidence intervals (CI). SIR was calculated by dividing the observed number of SPM cases by the expected number based on general population rates. Risks were considered significant when corresponding 95% CI did not include the null value. Age-adjusted incidence rates were used for comparison to exclude the potential confounding of the differences in age distributions. The analysis was performed in SEER*Stat version 8.3.5 (http://seer.cancer.gov/seerstat/). Multivariable Cox regression was performed to identify factors affected OS in patients with SPM after diagnosis of MA. A *P*-value <0.05 was considered significant. Analysis was performed using SPSS (Release 23.0, SPSS Inc, IL, U.S.A.). The prognostic nomogram that included all significant independent factors for OS in MA patients with SPM was presented in nomogram, which was performed in R (version 3.5.1; R Foundation).

## Results

### Risk of developing SPM in MA patients

The overall risk of SPM among MA patients was significantly higher than that in the general population (SIR: 1.09, 95% CI: 1–1.18, *P*<0.05). Specific sites where the risk of SPM increased included salivary gland, bone and joints, soft tissues including heart, brain, cranial nerves other nervous system, thyroid, acute non-lymphocytic leukemia and acute myeloid leukemia. Additionally, the risk of SPM in liver, breast and prostate was significantly decreased (Table 1).

**Table 1:** Risk of developing SPM in MA patients

Site of SPM	Observed	Expected	SIR	95% CI
All sites	586	538.97	1.09^1^	1–1.18
All solid tumors	511	480.12	1.06	0.97–1.16
Oral cavity and pharynx	14	15.5	0.9^2^	0.49–1.52
Digestive system	79	92	0.86^3^	0.68–1.07
Respiratory system	59	73.77	0.8	0.61–1.03
Bones and joints	10	1.21	8.3^1^	3.98–15.26
Soft tissue including heart	12	3.58	3.35^1^	1.73–5.86
Skin excluding Basal and Squamous	35	30.14	1.16	0.81–1.62
Breast	48	76.75	0.63^1^	0.46–.83
Female genital system	30	31.15	0.96	0.65–1.37
Male genital system	55	89.81	0.61^1,4^	0.46–0.8
Urinary system	35	40.34	0.87	0.6–1.21
Eye and orbit	1	1.05	0.95	0.02–5.3
Brain	100	8.09	12.36^1^	10.05–15.03
Cranial nerves other nervous system	8	0.48	16.53^1^	7.14–32.58
Endocrine system	24	14.76	1.63^1,5^	1.04–2.42
Lymphoma	26	26.84	0.97	0.63–1.42
Leukemia	30	14.68	2.04^1,6^	1.38–2.92
Mesothelioma	2	1.08	1.85	0.22–6.68
Kaposi’s sarcoma	2	2.39	0.84	0.1–3.02
Miscellaneous	12	9.01	1.33	0.69–2.33

^1^ *P*<0.05. ^2^ Risk of SPM in salivary gland significantly increased (SIR: 4.08, 95% CI: 1.5-8.88). ^3^ Risk of SPM in liver significantly decreased (SIR: 0.15, 95% CI: 0–0.86). ^4^ Risk of SPM in prostate significantly decreased (SIR: 0.57, 95% CI: 0.42–0.76). ^5^ Risk of SPM in thyroid significantly increased (SIR: 1.72, 95% CI: 1.1–2.55). ^6^ Risk of SPM in acute non-lymphocytic leukemia (SIR: 3.43, 95% CI: 1.96–5.57) and acute myeloid leukemia (SIR: 3.71, 95% CI: 2.07–6.11) significantly increased.

### Effect of age at diagnosis, gender and latency on the site-specific risk of SPM in MA patients

We further validated the effect of age at diagnosis, gender and latency (interval from the diagnosis of MA to that of SPM) on risk of developing SPM among MA patients. Based on the age at MA diagnosis, patients were divided into three groups: 0–29, 30–59 and ≥60 years. Overall risk of SPM in patients aged ≤29 and 30–59 years significantly increased (4.34- and 1.41-fold respectively). Whereas patients aged ≥60 years had a significantly decreased risk of SPM. As stratified by gender, the overall risk of SPM in males and females did not change among MA patients. The detailed site-specific risk of SPM in each group is shown in Table 2.

**Table 2:** Effect of age at diagnosis, gender on the site-specific risk of SPM in MA patients

Site of second malignancy	Age at diagnosis			Gender	
	0–29 years SIR, 95%	30–59 years SIR, 95%	≥60 years SIR, 95%	Male SIR, 95%	Female SIR, 95%
All sites	4.34 (3.33–5.55)^1^	1.41 (1.25–1.57)^1^	0.7 (0.61–0.81)^1^	1.07 (0.96–1.2)	1.11 (0.97–1.25)
All solid tumors	5.45 (4.13–7.06)^1^	1.4 (1.24–1.58)^1^	0.66 (0.57–0.76)^1^	1.04 (0.92–1.16)	1.1 (0.96–1.25)
Oral cavity and pharynx	11.87 (2.45–34.7)^1,2^	1.06 (0.46–2.08)	0.39 (0.08–1.14)	0.68 (0.29–1.34)	1.62 (0.59–3.52)^16^
Digestive system	4.68 (0.97–13.69)^3^	1.15 (0.82–1.58)^6^	0.65 (0.46–0.89)^1^	0.75 (0.55–1.01)^12^	1.05 (0.73–1.45)
Respiratory system	0 (0–7.38)	1.57 (1.09–2.18)^1,7^	0.47 (0.3–0.7)^1,10^	0.71 (0.49–0.99)^1^	0.96 (0.62–1.42)
Bones and joints	13.66 (5.01–29.73)^1^	7.88 (2.15–20.17)^1^	0 (0–14.27)	8.1 (2.97–17.63)^1^	8.61 (2.35–22.04)^1^
Soft tissue including heart	12.22 (4.48–26.59)^1^	3.47 (1.27–7.55)^1^	0 (0–2.72)	3.19 (1.28–6.57)^1^	3.62 (1.18–8.45)^1^
Skin excluding Basal and Squamous	2.74 (0.89–6.39)	1.16 (0.7–1.81)	0.92 (0.46–1.65)	1.43 (0.93–2.09)	0.75 (0.34–1.43)
Breast	5.99 (1.23–17.49)^1^	0.57 (0.36–0.84)^1^	0.62 (0.38–0.95)^1^	3.13 (0.38–11.32)	0.6 (0.44–0.81)^1^
Female genital system	2.28 (0.28–8.24)	0.92 (0.52–1.52)	0.93 (0.49–1.58)	0	0.96 (0.65–1.37)
Male genital system	0.6 (0.02–3.36)	0.76 (0.46–1.18)	0.56 (0.39–0.77)^1,11^	0.61 (0.46–0.8)^1,13^	0
Urinary system	3.11 (0.08–17.33)	1.01 (0.55–1.7)	0.76 (0.47–1.18)	0.91 (0.6–1.31)	0.74 (0.3–1.53)
Eye and orbit	16.17 (0.41–90.12)^4^	0 (0–7.33)	0 (0–7.6)	0 (0–5.74)	2.45 (0.06–13.66)
Brain	19.17 (12.01–29.02)^1^	17.47 (13.57–22.15)^1^	3.27 (1.57–6.02)^1^	10.58 (7.94–13.8)^1^	15.4 (11.27–20.54)^1^
Cranial nerves other nervous system	27.91 (5.75–81.55)^1^	16.04 (4.37–41.07)^1^	7.87 (0.2–43.87)	18.28 (5.94-42.67)^1^	14.26 (2.94–41.67)^1^
Endocrine system	1.09 (0.13–3.95)	2.06 (1.26–3.17)^1,8^	0.62 (0.08–2.26)	2.67 (1.38–4.66)^1,14^	1.17 (0.6–2.04)
Lymphoma	0.79 (0.1–2.85)	0.67 (0.29–1.32)	1.3 (0.74–2.11)	1.01 (0.59–1.62)	0.9 (0.41–1.7)
Leukemia	2.4 (0.49–7.01)^5^	3.23 (1.88–5.17)^1,9^	1.22 (0.59–2.25)	1.98 (1.19–3.09)^1,15^	2.16 (1.08–3.87)^1^
Mesothelioma	0 (0–664.97)	3.54 (0.09–19.75)	1.26 (0.03–7.01)	1.14 (0.03–6.34)	4.92 (0.12–27.4)
Kaposi’s sarcoma	0 (0–15.15)	0 (0–1.85)	13.17 (1.59–47.56)^1^	0.85 (0.1–3.07)	0 (0–85.97)
Miscellaneous	0 (0–36.54)	1.72 (0.56–4.01)	1.17 (0.47–2.4)	1.85 (0.89–3.4)	0.56 (0.07–2.01)

^1^ *P*<0.05. ^2^ Risk of SPM in salivary gland significantly increased (SIR: 29.88, 95% CI: 6.16–87.33). ^3^ Risk of SPM in colon, rectum and anus (SIR: 8.05, 95% CI: 1.66–23.53), especially in descending colon (SIR: 56.93, 95% CI: 1.44–317.19) significantly increased. ^4^ Risk of SPM in eye and orbit-melanoma significantly increased (SIR: 39.98, 95% CI: 1.01–222.73). ^5^ Risk of SPM in acute non-lymphocytic leukemia (SIR 7.21, 95% CI 1.49-21,08) and acute myeloid leukemia (SIR: 8.37, 95% CI: 1.73–24.47) significantly increased. ^6^ Risk of SPM in stomach significantly increased (SIR: 3.19, 95% CI: 1.46–6.05). ^7^ Risk of SPM in lung and bronchus significantly increased (SIR: 1.68, 95% CI 1.15–2.35). ^8^ Risk of SPM in thyroid significantly increased (SIR: 2.14, 95% CI: 1.31–3.31). ^9^ Risk of SPM in acute non-lymphocytic leukemia (SIR: 4.7, 95% CI: 2.03–9.26), acute myeloid leukemia (SIR: 4.69, 95% CI: 1.89–9.66) and chronic myeloid leukemia (SIR: 4.42, 95% CI: 1.2–11.31) significantly increased. ^10^ Risk of SPM in lung and bronchus significantly decreased (SIR: 0.46, 95% CI: 0.29–0.7). ^11^ Risk of SPM in prostate significantly decreased (SIR: 0.56, 95% CI: 0.39–0.78). ^12^ Risk of SPM in liver significantly decreased (SIR: 0, 95% CI: 0–0.7). ^13^ Risk of SPM in prostate significantly decreased (SIR: 0.57, 95% CI: 0.42–0.76). ^14^ Risk of SPM in thyroid significantly increased (SIR: 2.97, 95% CI: 1.54–5.19). ^15^ Risk of SPM in acute non-lymphocytic leukemia (SIR: 4.23, 95% CI: 2.18–7.38) and acute myeloid leukemia (SIR: 4.48, 95% CI: 2.23–8.01) significantly increased. ^16^ Risk of SPM in salivary gland significantly increased (SIR: 5.26, 95% CI: 1.08–15.38).

Median latency and corresponding interquartile range (IQR) were 22 months and 80 months, respectively. According to latency, patients were divided into five groups: ≤11, 12–35, 36–59, 60–119 and ≥120 months. Patients in the group of latency at 36–59, 60–119 and ≥120 months carried significantly increased overall risk of SPM. Patients in the group of latency at ≤11 and 12–35 months did not have significant change in the overall risk of SPM. Site-specific risk of SPM in each group is shown in Table 3.

**Table 3:** Effect of latency on the site-specific risk of SPM in MA patients

Site of second malignancy	≤11months SIR, 95%	12–35 months SIR, 95%	36–59 months SIR, 95%	60–119 months SIR, 95%	≥120 months SIR, 95%
All sites	1.13 (1–1.28)	1 (0.82–1.21)	1.62 (1.26–2.05)^1^	1.58 (1.3–1.91)^1^	1.23 (1.06–1.41)^1^
All solid tumors	1.04 (0.91–1.19)	0.97 (0.78–1.19)	1.64 (1.26–2.1)^1^	1.57 (1.27–1.91)^1^	1.24 (1.06–1.44)^1^
Oral cavity and pharynx	0.3 (0.04–1.1)	0 (0–1.21)	0.79 (0.02–4.42)	0.98 (0.12–3.56)	2.32 (1.11–4.26)^1,11^
Digestive system	0.57 (0.37–0.84)^1,2^	0.79 (0.43–1.33)	1.15 (0.5–2.27)	1.33 (0.74–2.19)	0.98 (0.63–1.45)
Respiratory system	1.2 (0.87–1.61)	0.89 (0.47–1.52)	0.71 (0.19–1.83)	0.9 (0.39–1.78)	0.85 (0.47–1.4)
Bones and joints	0 (0–12.66)	18.98 (5.17–48.6)^1^	23.14 (4.77–67.62)^1^	8.34 (1.01–30.12)^1^	2.47 (0.06–13.76)
Soft tissue including heart	1.72 (0.21–6.2)	4.58 (0.95–13.4)	6.27 (0.76–22.64)	3.65 (0.44–13.2)	2.51 (0.52–7.34)
Skin excluding Basal and Squamous	1.46 (0.8–2.44)	0.91 (0.3–2.13)	1.94 (0.63–4.52)	1.6(0.64–3.29)	0.85 (0.39–1.62)
Breast	0.43 (0.21–0.77)^1^	0.96 (0.51–1.64)	0.77 (0.25–1.79)	1.13 (0.6–1.94)	0.49 (0.26–0.84)^1^
Female genital system	1.01 (0.5–1.8)	0.36 (0.04–1.28)	1.13 (0.23–3.29)	0.87 (0.24–2.24)	1.46 (0.82–2.41)
Male genital system	0.5 (0.31–0.76)^1,3^	0.46 (0.2–0.9)^1,7^	1.43 (0.65–2.71)	0.88 (0.4–1.68)	0.84 (0.51–1.3)
Urinary system	1.94 (1.36–2.67)^1,4^	1.01 (0.44–2)	1.31 (0.36–3.37)	1.03 (0.34–2.41)	0.65 (0.26–1.34)
Eye and orbit	0 (0–8.98)	0 (0–17.95)	0 (0–41.59)	0 (0–25.97)	3.21 (0.08–17.87)
Brain	11.48 (7.86–16.21)^1^	8.49 (4.52–14.52)^1^	19.78 (11.07–32.62)^1^	20.56 (13.43–30.12)^1^	16.18 (11.56–22.04)^1^
Cranial nerves other nervous system	21.95 (4.53–64.15)^1^	0 (0–40.73)	20.1 (0.51–112.01)	11.98 (0.3–66.77)	38.17 (14.01–83.08)^1^
Endocrine system	2.82 (1.29–5.35)^1,5^	2.11 (0.68–4.92)	1.55 (0.19–5.6)	2.14 (0.69–4.99)	1.43 (0.65–2.71)
Lymphoma	0.72 (0.29–1.47)	1.61 (0.7–3.18)	1.29 (0.27–3.78)	0.51 (0.06–1.83)	1.07 (0.49–2.02)
Leukemia	2.89 (1.71–4.57)^1,6^	1.77 (0.57–4.12)^8^	2.44 (0.5–7.14)^9^	5.52 (2.76–9.88)^1,10^	1.69 (0.68–3.49)
Mesothelioma	3.42 (0.41–12.34)	0 (0–17.29)	0 (0–46.42)	0 (0–29.08)	0 (0–15.05)
Kaposi’s sarcoma	3.93 (0.48–14.19)	0 (0–7.54)	0 (0–11.53)	0 (0–7.12)	0 (0–5.54)
Miscellaneous	3.18 (1.78–5.24)^1^	0.58 (0.01–3.22)	0 (0–5.32)	0 (0–3.24)	0.95 (0.11–3.42)

^1^ *P*<0.05. ^2^ Risk of SPM in colon and rectum (SIR: 0.59, 95% CI: 0.33–0.98) and pancreas (SIR: 0.17, 95% CI: 0–0.95) significantly decreased. ^3^ Risk of SPM in prostate significantly decreased (SIR: 0.44, 95% CI: 0.27–0.69). ^4^ Risk of SPM in kidney significantly increased (SIR: 4.01, 95% CI: 2.57–5.96). ^5^ Risk of SPM in thyroid significantly increased (SIR: 3.05, 95% CI: 1.4–5.8). ^6^ Risk of SPM in chronic lymphocytic leukemia significantly increased (SIR: 5.62, 95% CI: 3.14–9.27). ^7^ Risk of SPM in prostate significantly decreased (SIR: 0.42, 95% CI: 0.17–0.87). ^8^ Risk of SPM in acute non-lymphocytic leukemia significantly increased (SIR: 4.55, 95% CI: 1.24–11.64). ^9^ Risk of SPM in acute non-lymphocytic leukemia significantly increased (SIR: 5, 95% CI: 1.03–14.61). ^10^ Risk of SPM in chronic lymphocytic leukemia (SIR: 6.16, 95% CI: 1.68–15.77), acute non-lymphocytic leukemia (SIR: 9.28, 95% CI: 3.41–20.2) and acute myeloid leukemia (SIR: 10.75, 95% CI: 3.94–23.39) significantly increased. ^11^ Risk of SPM in salivary gland significantly increased (SIR: 11.05, 95% CI: 3.59–25.8).

### Characteristics of MA patients with SPM

A total of 1181 patients with SPM after diagnosis of MA were recorded in SEERStat by case listing function. Characteristics of these patients are shown in Table 4. The median age for MA patients with SPM was 52 years old. A total of 88.45% (1045/1181) of them were whites. According to WHO grading system, 45.47% (537/1181) patients had glioblastoma (Grade IV astrocytoma); 51.23% (605/1181) of MA patients with SPM underwent surgery resection on astrocytoma; 94.75% (1119/1181) of patients developed two primaries, as well as 5.24% (62/1181) of them developed at least three primaries. Median OS was 48 months for this cohort.

**Table 4:** Characteristics of MA patients with SPM

	Patients, *n*=1181
Age, years, median (IQR)	52 (28)
Gender	
Male, *n* (%)	500 (42.34)
Female, *n* (%)	681 (57.66)
Race	
White, *n* (%)	1045 (88.48)
Black, *n* (%)	70 (5.93)
Others, *n* (%)	66 (5.59)
Marital status	
Married, *n* (%)	715 (60.54)
Unmarried, *n* (%)	432 (36.58)
Unknown, n (%)	34 (2.88)
WHO grade	
Specified low-grade astrocytic tumors, *n* (%)	150 (12.7)
AA, *n* (%)	160 (13.55)
Glioblastoma, *n* (%)	537 (45.47)
Unknown, *n* (%)	334 (28.28)
Surgery of first primary site	
Yes, *n* (%)	605 (51.23)
No, *n* (%)	135 (11.43)
Unknown, *n* (%)	441 (37.34)
Latency, months, median (IQR)	22 (80)
Number of primaries (including astrocytoma)	
2 primaries, *n* (%)	1119 (94.75)
3 primaries, *n* (%)	52 (4.4)
4 primaries, *n* (%)	9 (0.76)
5 primaries, *n* (%)	1 (0.08)
OS, months, median (IQR)	48 (128)

### Factors affecting the OS in MA patients with SPM

As shown in Table 5, patients with age 30–59 years (vs ≤29 years; HR: 2.241; 95% CI: 1.716–2.926; *P*<0.001), aged ≥60 years (vs ≤29 years; HR: 4.494; 95% CI: 3.358–6.015; *P*<0.001), unmarried people (vs married; HR: 1.358; 95% CI: 1.159–1.591; *P*<0.001), WHO grade as glioblastoma (vs specified low-grade astrocytic tumors; HR: 2.072; 95% CI: 1.54–2.787; *P*<0.001); moderately differentiated cancer tissues (vs well-differentiated; HR: 1.524; 95% CI: 1.028–2.259; *P*=0.036); poorly differentiated cancer tissues (vs well-differentiated; HR: 2.299; 95% CI: 1.444–3.658; *P*<0.001) and undifferentiated cancer tissues (vs well-differentiated; HR: 2.176; 95% CI: 1.432–3.305; *P*<0.001) were significantly associated with an increased mortality. Blacks (vs whites; HR: 0.627; 95% CI: 0.455–0.865; *P*=0.004); latency at 12–35 months (vs ≤11 months; HR: 0.345; 95% CI: 0.282–0.422; *P*<0.001), latency at 36–59 months (vs ≤11 months; HR: 0.197; 95% CI: 0.153–0.253; *P*<0.001), latency at 60–119 months (vs ≤11 months; HR: 0.132; 95% CI: 0.103–0.169; *P*<0.001) and latency ≥120 months (vs ≤11 months; HR: 0.063; 95% CI: 0.048–0.083; *P*<0.001) were significantly associated with a decreased mortality.

**Table 5:** Multivariable Cox regression for OS among MA patients with SPM

Parameters	Univariate analysis		Multivariate analysis	
	HR (95% CI)	*P*-value	HR (95% CI)	*P*-value
Age at diagnosis				
≤29	1 [Reference]		1 [Reference]	
30–59	2.594 (2.049–3.282)	<0.001	2.241 (1.716–2.926)	<0.001
≥60	8.631 (6.74–11.052)	<0.001	4.494 (3.358–6.015)	<0.001
Gender				
Female	1 [Reference]			
Male	1.34 (1.169–1.537)	<0.001	/	/
Race				
White	1 [Reference]		1 [Reference]	
Black	0.7 (0.518–0.946)	0.02	0.627 (0.455–0.865)	0.004
Others	0.776 (0.573–1.052)	0.102	1.019 (0.747–1.391)	0.904
Marital status				
Married	1 [Reference]		1 [Reference]	
Unmarried	0.682 (0.59–0.787)	<0.001	1.358 (1.159–1.591)	<0.001
Unknown	0.792 (0.526–1.192)	0.264	0.872 (0.576–1.318)	0.515
WHO grade				
Low-grade astrocytic tumors, Grade II	1 [Reference]		1 [Reference]	
AA, Grade III	1.817 (1.349–2.448)	<0.001	1.228 (0.861–1.751)	0.257
Glioblastoma, Grade IV	5.312 (4.133–6.829)	<0.001	2.072 (1.54–2.787)	<0.001
Unknown	1.241 (0.95–1.62)	0.113	1.033 (0.779–1.368)	0.823
Differentiated grade				
Grade I, well differentiated	1 [Reference]			
Grade II, moderately differentiated	1.65 (1.126–2.42)	0.01	1.524 (1.028–2.259)	0.036
Grade III, poorly differentiated	2.879 (1.854–4.471)	<0.001	2.299 (1.444–3.658)	<0.001
Grade IV, undifferentiated	3.684 (2.569–5.282)	<0.001	2.176 (1.432–3.305)	<0.001
Unknown	3.623 (2.55–5.148)	<0.001	2.076 (1.403–3.071)	<0.001
SEER stage				
Localized	1 [Reference]		1 [Reference]	
Regional	1.498 (1.159–1.936)	0.002	1.284 (0.99–1.666)	0.06
Distant	2.228 (0.922–5.385)	0.075	2.192 (0.897–5.357)	0.085
Unknown	0.569 (0.487–0.665)	<0.001	1.291 (1.091–1.527)	0.003
Surgery of first primary site				
Yes	1 [Reference]			
No	1.028 (0.823–1.284)	0.807	/	/
Unknown	0.559 (0.477–0.655)	<0.001	/	/
Latency				
≤11 months	1 [Reference]		1 [Reference]	
12–35 months	0.354 (0.292–0.429)	<0.001	0.345 (0.282–0.422)	<0.001
36–59 months	0.206 (0.162–0.261)	<0.001	0.197 (0.153–0.253)	<0.001
60–119 months	0.105 (0.084–0.133)	<0.001	0.132 (0.103–0.169)	<0.001
≥120 months	0.05 (0.039–0.063)	<0.001	0.063 (0.048–0.083)	<0.001
Number of primary tumors				
2	1 [Reference]			
At least 3	0.773 (0.576–1.038)	0.087	/	/

### Prognostic nomogram for OS in MA patients with SPM

**Figure 1:**
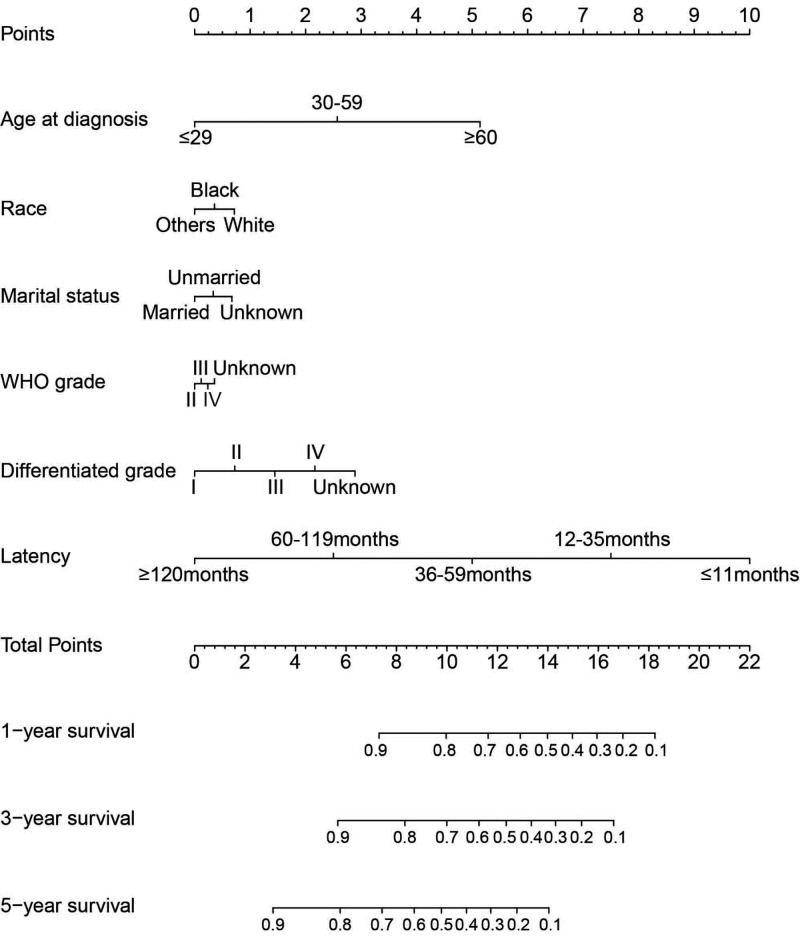
Prognostic nomogram for OS in MA patients with SPM

**Figure 2:**
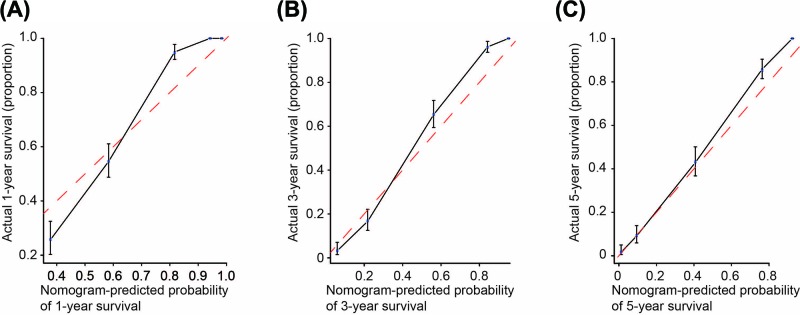
Calibration curves for predicting patient survival at 1-, 3- and 5-years

The prognostic nomogram that included all significant independent indicators for OS in MA patients with SPM is shown in Figure 1. The C-index for OS prediction was 0.851. The calibration plot for the probability of survival at 1, 3 and 5 years after diagnosis showed an optimal agreement between the prediction by nomogram and actual observation (Figure 2).

## Discussion

Given the elevated incidence and survival of MA patients [18], there is an increasing focus on long-term effects of cancer and its related treatment, especially the risk of developing SPM. This population-based cohort study of MA patients strongly indicated that there was a significantly elevated risk of tumor in brain (SIR: 12.36, 95% CI: 10.05–15.03) and cranial nerves (SIR: 16.53, 95% CI: 7.14–32.58). One explanation could be a known consequence of cranial irradiation [19,20]. Another consideration is whether these brain and cranial nerves tumor diagnoses are metastases from the primary malignancy. As stated in the ‘Methods’ section, we used SEER definition to identify patients with SPM, which could exclude metastatic and recurrent tumors from SPM counts. Previous study evaluated the validity of SPM in public database with a revaluation of diagnoses, which showed that 98% of the second primary diagnoses were correct [21]. Therefore, we can almost get rid of the concern.

It was also notable that patients with astrocytoma were at a significantly higher risk of developing malignancy in bones and joints (SIR: 8.3, 95% CI: 3.98–15.26), soft tissue including heart (SIR: 3.35, 95% CI: 1.73–5.86), leukemia (SIR: 2.04, 95% CI: 1.38–2.92) and thyroid (SIR: 1.72, 95% CI: 1.1–2.55). Explanations for why patients with astrocytoma had higher risk of developing above-mentioned malignancies are not quite clear. It is usually attributed to treatment-related factors including radiation and chemotherapy. Temozolomide, widely used for treating high-grade astrocytoma, was reported to cause leukemia [22]. Except for that, underlying genetic cause is one of the critical factors. Isocitrate dehydrogense (IDH) mutation was associated with the development and progression of various malignancies, including astrocytoma, leukemia, malignancy of soft tissue and bones [23–26]. FGFR gene fusions were identified in thyroid malignancy and glioblastoma [27]. Fifteen percent of patients with glioblastomas carried PDGFRA amplification, which was also found in soft tissue malignancy [28,29].

The current study found that in the whole cohort of MA patients, the risk of developing prostate cancer declined significantly (SIR: 0.57, 95% CI: 0.42–0.76), especially in the subgroup of patients aged ≥60 years, patients within 11 and 12–35 months after MA diagnosis. As well as a declined risk of developing breast cancer in whole cohort of astrocytoma patients (SIR: 0.63, 95% CI: 0.46–0.83), especially in the subgroup of female patients, patients aged 30–59 and ≥60 years. Malmer et al. [30] reported a significantly decreased risk of astrocytoma patients to develop breast cancer, which is consistent with our study. Malmer et al.’s [30] study also found a declined risk of developing colorectal cancer in the cohort of astrocytoma patients, which was evident in our study merely in patients within 11 months after MA diagnosis (SIR: 0.59, 95% CI: 0.33–0.98). However, reasons for why MA patients had lower risk of developing the aforementioned malignancies are still unknown, which needs further investigation.

Identification of prognostic factors among MA patients who developed SPM would favor for outcome prediction and treatment decision. Our study found that unmarried people had a higher risk of mortality, which was also proved in other solid tumors [31]. It revealed a phenomenon that social support was a critical factor for cancer patients’ survival and more intensive social support was necessary. Our study also found that among MA patients who developed SPM, the age, race, WHO grade, differentiated grade of cancer tissues were prognostic factors for OS, which was consistent with previous studies [32,33]. Our analysis revealed that longer the latency, higher the risk of developing SPM in MA patients. Whereas, shorter the latency, poorer the OS in MA patients with SPM. Furthermore, the median interval from MA diagnosis to that of SPM was 22 months, which indicated that during this period intensive surveillance was recommended.

The present study carried several strengths. First, it was the first study which reported the risk of SPM in MA patients. Second, it was based on a population with large sample size and long-term follow-up. Third, it provided strong evidence for the development of guidelines of surveillance of MA survivors. We analyzed the risk of developing SPM in subgroup of patients with different age, gender and interval from diagnosis of MA to that of SPM, which provided detailed information regarding patients’ prognosis and focusing clinical follow-up in each subgroup. For example, our study showed that patients aged ≤29 years had significantly increased risk of developing colon cancer. If a patient understands his/her risk for developing the aforementioned cancer, he/she may choose to alter her treatment to be more aggressive in hopes of reducing his/her risk. He/she may also need to be counselled regarding preventable risk factors, such as weight gain and poor dietary habits. Lack of variables such as radiation, chemotherapy and genetic make-up was the main limitation of the study.

## Conclusion

In conclusion, we observed that patients survived from MA were at a significantly higher overall risk of developing SPM. Specific sites where the risk of SPM was increased including brain, cranial nerves thyroid, acute non-lymphocytic leukemia and acute myeloid leukemia. Additionally, excess risks varied with age, gender and interval from diagnosis of MA to that of SPM. Age, race, marital status, WHO grade, differentiated grade of cancer tissues, latency were independent predictors of OS in MA patients with SPM. The results of our study suggested the need for closer surveillance of at-risk MA patients for the development of SPM and for further research into the molecular pathways underlying the occurrence of SPM.

## Data Availability

The data used to support the findings of the present study are available from the corresponding author upon request.

## Abbreviations

AA: anaplastic astrocytoma
CI: confidence interval
DA: diffuse astrocytoma
FGFR: fibroblast growth factor receptor
HR: hazard ratio
MA: malignant astrocytoma
OS: overall survival
PDGFRA: platelet-derived growth factor receptor alpha
SEER: Surveillance, Epidemiology and End Results
SIR: standardized incidence ratio
SPM: second primary malignancy
